# Hologram QSAR Models of a Series of 6-Arylquinazolin-4-Amine Inhibitors of a New Alzheimer’s Disease Target: Dual Specificity Tyrosine-Phosphorylation-Regulated Kinase-1A Enzyme

**DOI:** 10.3390/ijms16035235

**Published:** 2015-03-06

**Authors:** Felipe Dias Leal, Camilo Henrique da Silva Lima, Ricardo Bicca de Alencastro, Helena Carla Castro, Carlos Rangel Rodrigues, Magaly Girão Albuquerque

**Affiliations:** 1 Instituto de Química, Laboratório de Modelagem Molecular (LabMMol), Universidade Federal do Rio de Janeiro (UFRJ), 21949-900 Rio de Janeiro, RJ, Brazil; E-Mails: camilolima@iq.ufrj.br (C.H.S.L.); bicca@iq.ufrj.br (R.B.A.); 2 Instituto de Biologia, Laboratório de Antibióticos, Bioquímica, Ensino e Modelagem Molecular (LABiEMol), Universidade Federal Fluminense (UFF), 24210-130 Niterói, RJ, Brazil; E-Mail: hcastrorangel@yahoo.com.br; 3 Faculdade de Farmácia, Laboratório de Modelagem Molecular & 3D-QSAR (ModMolQSAR), Universidade Federal do Rio de Janeiro (UFRJ), 21941-590 Rio de Janeiro, RJ, Brazil; E-Mail: rangel@pharma.ufrj.br

**Keywords:** Alzheimer’s disease, molecular hologram, HQSAR, molecular modeling, DYRK1A inhibitors

## Abstract

Dual specificity tyrosine-phosphorylation-regulated kinase-1A (DYRK1A) is an enzyme directly involved in Alzheimer’s disease, since its increased expression leads to β-amyloidosis, Tau protein aggregation, and subsequent formation of neurofibrillary tangles. Hologram quantitative structure-activity relationship (HQSAR, 2D fragment-based) models were developed for a series of 6-arylquinazolin-4-amine inhibitors (36 training, 10 test) of DYRK1A. The best HQSAR model (*q*^2^ = 0.757; SE_cv_ = 0.493; *R*^2^ = 0.937; SE = 0.251; *R*^2^pred = 0.659) presents high goodness-of-fit (*R*^2^ > 0.9), as well as high internal (*q*^2^ > 0.7) and external (*R*^2^pred > 0.5) predictive power. The fragments that increase and decrease the biological activity values were addressed using the colored atomic contribution maps provided by the method. The HQSAR contribution map of the best model is an important tool to understand the activity profiles of new derivatives and may provide information for further design of novel DYRK1A inhibitors.

## 1. Introduction

Alzheimer’s disease (AD) is a neurodegenerative disorder that accounts for 60% to 70% of all cases of dementia and consists of loss or impairment of memory and other cognitive skills [1]. In brains of patients with AD, at the macroscopic level, it is observed severe brain atrophy; while at the microscopic level, it can be observed amyloid plaques, neurofibrillary tangles, and extensive neuronal loss [2]. The major protein component of plaques is the beta-amyloid (Aß) peptide, derived from the amyloid precursor protein (APP), which is present as insoluble aggregates [3]. This peptide can be detected by immune-histochemical techniques as neuritic plaques (when aggregation occurs in β-sheet format) and diffuse plaques (when aggregation occurs in a non β-sheet format) [4]. Around the neuritic plaques, there is also an inflammatory process, involving hypertrophy and changes in morphology of glial cells and proliferation of astrocytes and microglia, which results in brain damage [5].

There is currently no cure for AD patients, but two main approved pharmacological strategies are available to help delay the condition’s development. The (acetyl- and/or butyryl) cholinesterase inhibitors (ChEIs) and glutamate *N*-methyl-d-aspartate (NMDA) receptor antagonists are used as combined or monotherapy. The ChEIs drugs such as tacrine, donepezil, galantamine (reversible inhibitor), and rivastigmine (pseudo-irreversible inhibitor) are used because this disease involves a cholinergic deficiency. The NMDA receptor antagonist memantine, the only drug from this class, is used because glutamate can act as an excitotoxin and cause neuronal death [6]. Other classes of drugs have been proposed, such as antioxidants, estrogens, statins, anti-inflammatory drugs, and Ginkgo biloba, but none of these has proven its clinical use. The discovery of new agents for the treatment of AD is important because the drugs currently used are not able to cure the disease, but only delay their advance [6].

The dual specificity kinases regulated by tyrosine (Tyr, Y) phosphorylation (DYRKs) are a family of eukaryotic kinases that belong to a superfamily known as CMGC kinases. The DYRK family contains five subtypes: 1A, 1B, 2, 3, and 4. However, only the DYRK1A gene is located within the human chromosome 21, more particularly, in the critical region of Down syndrome [7]. DYRK1A protein expression is widespread throughout the human body, but is particularly abundant in the cerebellum, olfactory bulb, and hippocampus. In addition, this protein has an up-regulation in the early stages of embryonic development, followed by gradual decrease in the later levels [8]. Although AD is a complex disease with several pathogenic mechanisms, this work focuses on the importance of the DYRK1A hyperexpression, since the increased expression of this enzyme leads to hyper-phosphorylation of Tau protein and APP, which results in high levels of Aß peptide (leading to β-amyloidosis) and aggregation of Tau protein, and subsequent formation of neurofibrillary tangles [9]. Due to the involvement of DYRK1A in the pathophysiological process of AD, this protein is recognized as a potential therapeutic target for this disease, which has led some research groups to synthesize and evaluate new compounds as potential inhibitors of this protein [10].

There are different classes of DYRK1A inhibitors, some of them are natural products or derivatives and other are synthetic compounds. Among the natural products, harmine, an alkaloid isolated from the South American plant *Banisteriopsis caapi*, and epigallocatechin gallate, a polyphenol present in green tea, were the first compounds shown to be potent and relatively selective inhibitors of DYRK1A [11]. Other natural products are quinalizarine [12]; flavonoids alcalinol A and B [13]; benzocoumarines [14]; and indolocarbazoles, such as staurosporine and rebeccamycin [15]. Among the synthetic compounds are: pirazolidine-3,5-diones [16]; meriolins [17]; meridianins [18], cromenoindoles [19]; and 6-arylquinazolin-4-amines [20]. All those compounds are still being tested *in vitro*, and no clinical tests have been conducted so far.

2D and 3D quantitative structure-activity relationship (QSAR) studies are widely employed to develop models, which are capable to explain the biological activity of a series of compounds and to predict the biological activity of new compounds [21,22,23,24,25,26]. 2D-QSAR methods use 2D-fragments and its physicochemical properties to generate predictive quantitative models. Examples of these methods are the fragment-based QSAR (FB-QSAR) [27,28] and hologram QSAR (HQSAR) [29].

As others 2D-QSAR methods, HQSAR is independent of the receptor (e.g., enzyme) structure and uses molecular holograms from 2D molecular fragmentation. In this 2D-QSAR method, each molecule is described by a molecular hologram called bin, which in turn is derived from molecular fragmentation and fragment arrangement, generating a molecular fingerprint. The descriptors used in HQSAR codify linear, branched or overlapped topological fragments, but additional 3D information, such as hybridization and chirality, may also be codified. The main advantage of this 2D-QSAR technique, over the current 3D-QSAR methods, is the fact that there is no need to generate the so-called “bioactive” conformations and molecular alignments. Only the compounds structures and their respective biological activity (or other properties) values are required for the application of this method [29].

In general, QSAR models can be classified as local or global [30]. A local model is derived from a small and similar set of chemical compounds, while a global model, from a chemically diverse large set [30]. Local models reflect the classical approach to QSAR [31], which are often used for drug design purposes when a common mode of action is known. Global models are often used for toxicity screening of pharmaceuticals for regulatory purposes [32].

Therefore, the main purpose of this work is to develop local HQSAR models for a series of 6-arylquinazolin-4-amine inhibitors of DYRK1A [20,33], which may be used to design novel and potent derivatives as potential drugs for the treatment of AD.

## 2. Results and Discussion

### HQSAR Model Development

At first, the hologram sizes were set as the prime numbers available in the HQSAR program in order to minimize the probability of bad fragment collisions. Then, maintaining the default fragment size values (4–7 atoms), the maximum number of components (NC) was set to fifteen, which is smaller than half the number of training set compounds (*N* = 36). Finally, various fragment distinction (FD) parameters were tested, obtaining sixteen different models (Table 1).

**Table 1:** Summary of the HQSAR statistical indexes for various fragment distinction (FD) parameters using the default fragment size (4–7 atoms) for the 6-arylquinazolin-4-amine derivatives (*N* = 36).

FD ^b^	Statistical Indexes ^a^					
	*q*^2^	*R*^2^	SE	SE_cv_	NC	HL
**A/B**	**0.732**	**0.847**	**0.373**	**0.493**	**3**	**61**
A/C	0.728	0.799	0.421	0.489	2	353
A/H	0.640	0.782	0.444	0.571	3	199
A/DA	0.697	0.896	0.323	0.551	6	59
B/C	0.711	0.841	0.380	0.512	3	53
B/H	0.727	0.824	0.400	0.498	3	59
**C/H**	**0.740**	**0.801**	**0.419**	**0.478**	**2**	**353**
C/DA	0.720	0.834	0.394	0.512	4	61
A/B/C	0.724	0.855	0.323	0.500	3	53
A/B/H	0.670	0.781	0.446	0.547	3	401
A/C/H	0.656	0.818	0.413	0.567	4	401
A/C/DA	0.721	0.842	0.394	0.511	4	61
B/C/Ch	0.711	0.841	0.380	0.512	3	53
A/B/C/H	0.691	0.777	0.443	0.521	2	353
**A/C/Ch/DA**	**0.742**	**0.876**	**0.341**	**0.491**	**4**	**257**
**A/B/C/Ch/DA**	**0.743**	**0.917**	**0.284**	**0.498**	**5**	**53**

^a^ *q*^2^, LOOcv (leave-one-out cross-validated) correlation coefficient; *R*^2^, non-cross-validated correlation coefficient; SE, non-cross-validated standard error; SEcv, cross-validated standard error; NC, optimal number of components; HL, hologram length; ^b^ FD, Fragment distinction parameters: atoms (A); bonds (B); connections (C); chirality (Ch); hydrogen (H) and H-bond donor/acceptor (DA) atoms. The four best models are in bold.

According to Table 2, all the HQSAR models were acceptable, since the lowest cross-validated correlation coefficient (*q*^2^) is 0.640. However, considering only models showing *q*^2^ values higher than 0.730, there were four best models, *i.e.*, **A/B/C/Ch/DA** (*q*^2^ = 0.743), **A/C/Ch/DA** (*q*^2^ = 0.742), **C/H** (*q*^2^ = 0.740), and **A/B** (*q^2^* = 0.732), which were used to evaluate the influence of fragment size on model quality.

In order to improve the previously calculated models, eight new templates were generated to each of the four best models, considering different fragment sizes, starting from two to twelve atoms, varying in four units each fragment (2–5, 3–6, 4–7, 5–8, 6–9, 7–10, 8–11, and 9–12 atoms). Only the statistical indexes obtained for the models using the **A/B/C/Ch/DA** (Table 2) and **A/B** (Table 3) parameters are shown, since the statistical indexes obtained for the models using the **C/H** and **A/C/Ch/DA** parameters did not show improvement. The fragment size variation improved the *q^2^* and *R^2^* values and minimizes the SE values, resulting in two best models (Table 2 and Table 3).

**Table 2:** Summary of the HQSAR statistical indexes for the influence of various fragment sizes (FS, 2–12 atoms) using the fragment distinction parameter **A/B/C/Ch/DA** for the 6-arylquinazolin-4-amine derivatives (*N* = 36).

FS	Statistical Indexes ^a^					
	*q*^2^	*R*^2^	SE	SE_cv_	NC	HL
2–5	0.734	0.855	0.362	0.491	3	401
3–6	**0.757**	**0.937**	**0.251**	**0.493**	**6**	**53**
4–7	0.743	0.917	0.284	0.498	5	53
5–8	0.751	0.883	0.331	0.483	4	53
6–9	0.738	0.871	0.347	0.496	4	61
7–10	0.732	0.920	0.282	0.518	6	53
8–11	0.681	0.906	0.302	0.556	5	151
9–12	0.642	0.804	0.421	0.570	3	151

^a^ *q*^2^, LOOcv (leave-one-out cross-validated) correlation coefficient; *R*^2^, non cross-validated correlation coefficient; SE, non cross-validated standard error; SEcv, cross-validated standard error; NC, optimal number of components; HL, hologram length. The best model is in bold.

**Table 3:** Summary of the HQSAR statistical indexes for the influence of various fragment sizes (FS, 2–12 atoms) using the fragment distinction parameter **A/B** for the 6-arylquinazolin-4-amine derivatives (*N* = 36).

FS	Statistical Indexes ^a^					
	*q*^2^	*R*^2^	SE	SE_cv_	NC	HL
2–5	0.737	0.848	0.372	0.488	3	61
3–6	0.717	0.858	0.359	0.507	3	83
4–7	0.732	0.847	0.373	0.493	3	61
5–8	0.713	0.839	0.382	0.510	3	61
6–9	0.719	0.848	0.377	0.513	4	61
7–10	**0.748**	**0.847**	**0.372**	**0.478**	**3**	**199**
8–11	0.724	0.848	0.371	0.500	3	401
9–12	0.705	0.829	0.394	0.517	3	83

^a^ *q*^2^, LOOcv (leave-one-out cross-validated) correlation coefficient; *R*^2^, non cross-validated correlation coefficient; SE, non cross-validated standard error; SEcv, cross-validated standard error; NC, optimal number of components; HL, hologram length. The best model is in bold.

The best model of the fragment distinction parameter **A/B/C/Ch/DA** contains 3–6 atoms per fragment (Table 2), while the best model of the fragment distinction parameter **A/B** contains 7–10 atoms per fragment (Table 3). It is worthy to note that the best model is the one containing five fragment distinction parameters (**A/B/C/Ch/DA**) and a fragment size of 3–6 atoms (Table 2), which means that the biological activity of this series of compounds seems to be better explained by a varied set of parameters in a fragment of reduced size. Thus, removing any of these parameters in the model leads to significant loss of information.

The Y-randomization test was carried out in order to analyze the robustness of the best models obtained (Table 2 and Table 3). In this test, the biological activity values were randomized and new HQSAR runs were performed (Table 4). According to Table 4, all models obtained by the Y-randomization test were very poor (the highest *q*^2^ value was 0.211) and this result reinforced the robustness of the original models, since there were low probability that the observed correlation occurred by chance.

**Table 4:** Summary of the HQSAR statistical indexes in the Y-randomization test using the default fragment size (4–7 atoms) for the 6-arylquinazolin-4-amine derivatives (*N* = 36).

FD ^b^	Statistical Indexes ^a^					
	*q*^2^	*R*^2^	SE	SE_cv_	NC	HL
**A/B**	0.143	0.396	0.694	0.827	2	353
A/C	0.117	0.722	0.502	0.895	6	59
A/H	0.058	0.381	0.703	0.867	2	199
A/DA	0.113	0.586	0.593	0.868	4	59
B/C	0.062	0.183	0.795	0.852	1	53
B/H	0.041	0.824	0.400	0.498	3	59
**C/H**	0.055	0.264	0.756	0.868	2	401
C/DA	0.089	0.202	0.785	0.840	1	53
A/B/C	0.211	0.713	0.510	0.846	6	61
A/B/H	0.044	0.351	0.719	0.873	2	401
A/C/H	0.045	0.359	0.715	0.872	2	353
A/C/DA	0.098	0.215	0.779	0.835	1	71
B/C/Ch	0.062	0.183	0.794	0.852	1	53
A/B/C/H	0.051	0.314	0.739	0.870	2	257
**A/C/Ch/DA**	0.106	0.222	0.776	0.832	1	71
**A/B/C/Ch/DA**	0.099	0.235	0.770	0.835	1	151

^a^ *q*^2^, LOO cross-validated correlation coefficient; *R*^2^, non-cross-validated correlation coefficient; SEcv, cross-validated standard error; SE, non-cross-validated standard error; NC, optimal number of components; HL, hologram length; ^b^ Fragment distinction parameters: atoms (A), bonds (B), connections (C), chirality (Ch), hydrogen (H) atoms, and donor/acceptor (DA) atoms.

After generation and internal validation of the best model, the external validation was carried out in order to access its ability to predict the biological activity values for the test set compounds, *i.e.*, those compounds excluded from the training set used for model generation. The predictive ability of the HQSAR model is expressed by predictive *R*^2^ values, which are similar to cross-validated *R*^2^ (*q*^2^), and calculated using Equation (1).

(1) $$r_{pred}^{2}=\frac{SD-PRESS}{SD}$$

**Figure 1:**
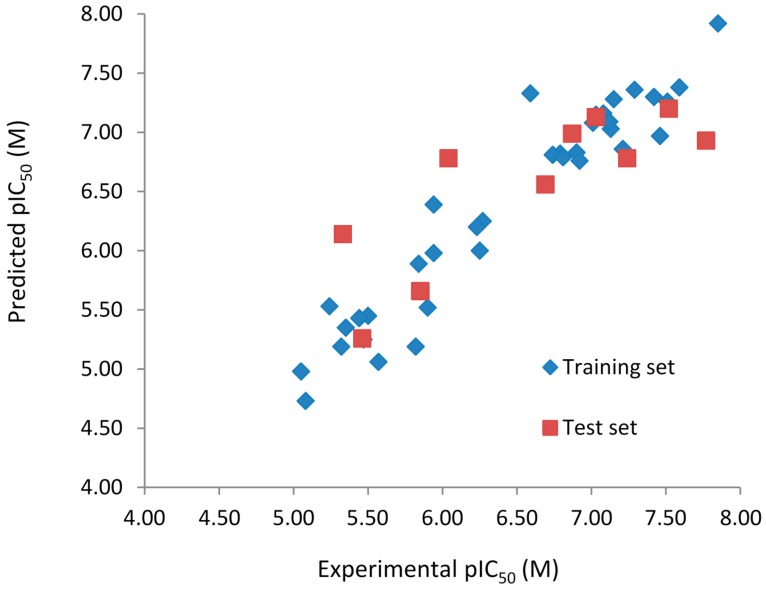
Experimental *vs.* predicted pIC_50_ values of the training (blue) and test (red) sets obtained using the best model with the fragment distinction parameters **A/B/C/Ch/DA**.

**Figure 2:**
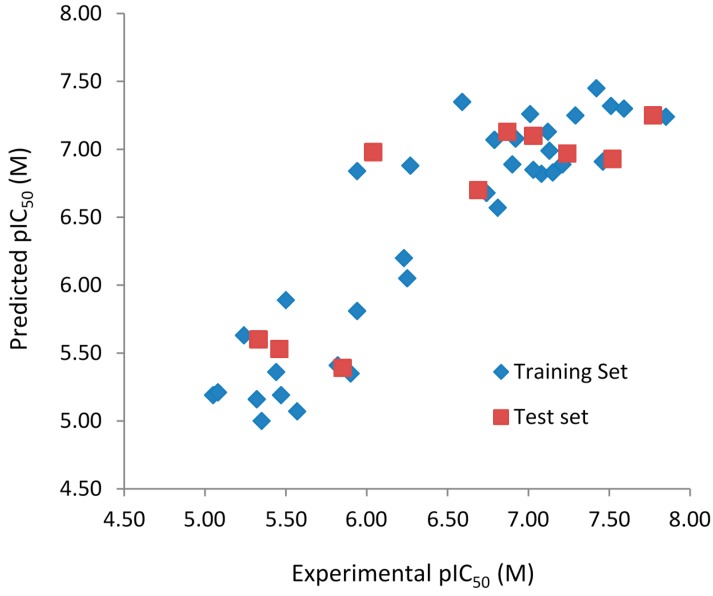
Experimental *vs.* predicted pIC_50_ values of the training (blue) and test (red) sets obtained using the best model with the fragment distinction parameters **A/B**.

The experimental (pIC_50Exp_) and predicted (pIC_50Pred_) biological activities, and residuals (pIC_50Exp_ − pIC_50Pred_) of the 6-arylquinazolin-4-amine derivatives obtained by the best HQSAR models from the fragment distinction parameters **A/B/C/Ch/DA** and **A/B** are reported in Table 5 and Table 6, respectively. The comparison plots between the pIC_50Exp_ and pIC_50Pred_ values of both training and test sets of the best HQSAR models from the fragment distinction parameters **A/B/C/Ch/DA** and **A/B** are shown in Figure 1 and Figure 2, respectively.

**Table 5:** Experimental pIC_50_ (Exp) and predicted pIC_50_ (Pred) biological activities, and residuals (Res = Exp − Pred) of the 6-arylquinazolin-4-amine derivatives using the best HQSAR model with the fragment distinction parameters **A/B/C/Ch/DA**.

# ^a^	Exp	Pred	Res	# ^a^	Exp	Pred	Res
**1**	7.21	6.86	0.35	**26**	7.29	7.36	−0.07
**2**	5.90	5.52	0.38	**27**	7.59	7.38	0.21
**3** *	5.46	5.26	0.20	**28**	6.81	6.79	0.02
**4**	5.24	5.53	−0.29	**29** *	6.04	6.78	−0.74
**5**	5.50	5.45	0.05	**30**	6.27	6.25	0.02
**6**	5.05	4.98	0.07	**31**	6.92	6.76	0.16
**7**	6.79	6.82	−0.03	**32 (*R*)** *	7.03	7.13	−0.10
**8**	5.35	5.35	0.00	**32 (*S*)** *	7.03	7.25	−0.22
**9**	6.74	6.81	−0.07	**33 (*R*)** *	6.87	6.99	−0.12
**10**	5.84	5.89	−0.05	**33 (*S*)** *	6.87	6.98	−0.11
**11** *	5.33	6.14	−0.81	**34 (*R*)** *	7.52	6.97	0.55
**12**	7.51	7.26	0.25	**34 (*S*)** *	7.52	6.97	0.55
**13**	7.42	7.30	0.12	**35**	7.12	7.09	0.03
**14**	5.94	6.39	0.45	**36** *	7.77	6.93	0.84
**15**	6.59	7.33	−0.74	**37**	5.94	5.98	0.04
**16**	7.46	6.97	0.49	**38**	6.25	6.00	0.25
**17**	7.08	7.16	−0.08	**39**	6.23	6.20	0.03
**18**	7.01	7.08	−0.07	**40**	5.44	5.43	0.01
**19**	7.13	7.03	0.10	**41**	5.47	5.25	0.22
**20** *	7.24	6.78	0.46	**42**	5.82	5.19	0.63
**21**	6.90	6.83	0.07	**43** *	5.85	5.66	0.19
**22**	7.03	7.15	−0.12	**44**	5.57	5.06	0.51
**23** *	6.69	6.56	0.13	**45**	5.31	5.19	0.13
**24**	7.85	7.92	−0.07	**46**	5.08	4.73	0.35
**25**	7.15	7.28	−0.13	-	-	-	-

^a^ Test set compounds are marked with an asterisk (*).

**Table 6:** Experimental pIC_50_ (Exp) and predicted pIC_50_ (Pred) biological activities, and residuals (Res = Exp − Pred) of the 6-arylquinazolin-4-amine derivatives using the best HQSAR model with the fragment distinction parameters **A/B**.

# ^a^	Exp	Pred	Res	# ^a^	Exp	Pred	Res
**1**	7.21	6.89	0.32	**26**	7.29	7.25	0.04
**2**	5.90	5.35	0.55	**27**	7.59	7.30	0.29
**3** *	5.46	5.53	−0.07	**28**	6.81	6.57	0.24
**4**	5.24	5.63	−0.39	**29** *	6.04	6.98	−0.94
**5**	5.50	5.89	−0.39	**30**	6.27	6.88	−0.61
**6**	5.05	5.19	−0.14	**31**	6.92	7.08	−0.16
**7**	6.79	7.07	−0.28	**32 (*R*)** *	7.03	7.10	−0.07
**8**	5.35	5.00	0.35	**32 (*S*)** *	7.03	7.10	−0.07
**9**	6.74	6.68	0.06	**33 (*R*)** *	6.87	7.13	−0.26
**10**	5.84	5.39	0.45	**33 (*S*)** *	6.87	7.13	−0.26
**11** *	5.33	5.60	−0.27	**34 (*R*)** *	7.52	6.93	0.59
**12**	7.51	7.32	0.19	**34 (*S*)** *	7.52	6.93	0.59
**13**	7.42	7.45	−0.03	**35**	7.12	7.13	−0.01
**14**	5.94	6.84	−0.90	**36** *	7.77	7.25	0.52
**15**	6.59	7.35	−0.76	**37**	5.94	5.81	0.13
**16**	7.46	6.91	0.55	**38**	6.25	6.05	0.20
**17**	7.08	6.82	0.26	**39**	6.23	6.20	0.03
**18**	7.01	7.26	−0.25	**40**	5.44	5.36	0.08
**19**	7.13	6.99	0.14	**41**	5.47	5.19	0.28
**20** *	7.24	6.97	0.27	**42**	5.82	5.41	0.41
**21**	6.90	6.89	0.01	**43** *	5.85	5.39	0.46
**22**	7.03	6.85	0.18	**44**	5.57	5.07	0.50
**23** *	6.69	6.70	−0.01	**45**	5.31	5.16	0.16
**24**	7.85	7.24	0.61	**46**	5.08	5.21	−0.13
**25**	7.15	6.83	0.32				

^a^ Test set compounds are marked with asterisk (*).

Both models do not contain outliers, defined as those compounds with residual values exceeding one logarithmic unit. However, the standard deviation (SD) of the residual values from the model with the fragment distinction parameters **A/B/C/Ch/DA** (SD = 0.322) is lower than the model with the fragment distinction parameters **A/B** (SD = 0.379), showing that the predicted pIC_50_ values are closer to the respective experimental ones. Each of the three compounds containing one chiral center (**32**, **33**, and **34**), modeled in both enantiomeric forms (*R* and *S*), presents identical or very close residual value, independent of the enantiomer and the model considered (Table 6 and Table 7), indicating that this chiral center has no relevance in the SAR study of this series of compounds. The correlation coefficient (*R*^2^_t_) and root-mean-square error (RMSE) calculated for the test are (*R*^2^_t_ = 0.654; RMSE = 0.484) for the **A/B/C/Ch/DA** model and (*R*^2^_t_ = 0.711; RMSE = 0.440) for the **A/B** model. These values support the statistical quality of both models. The *R*^2^_pred_ values for models **A/B/C/Ch/DA** (*R*^2^_pred_ = 0.659) and **A/B** (*R*^2^_pred_ = 0.743) are higher than 0.5, indicating that both models have acceptable prediction power.

**Figure 3:**
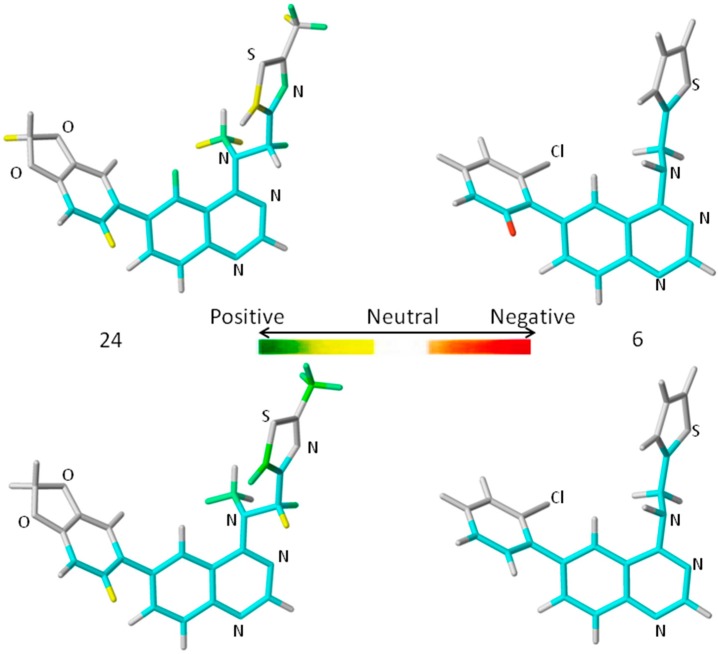
The HQSAR contribution maps of the most (**24**, **left**) and least (**6**, **right**) active compounds, according to the two best HQSAR models **A/B/C/Ch/DA** (**top**) and **A/B** (**bottom**). Color code: yellow-to-green, white, and orange-to-red represent positive, neutral, and negative contributions to the biological activity, respectively, and cyan represents the common backbone. The Cl, N, O, and S heteroatoms are labeled by element symbol, C and H atoms are not labeled.

A comprehensive analysis also involves the interpretation of the corresponding HQSAR colored diagrams (contribution maps) in which the colors represent positive (yellow-to-green), neutral (white), and negative (orange-to-red) contributions to the biological activity. Figure 3 shows the colored diagrams for the most (**24**) and least (**6**) active compounds for the two best models (**A/B/C/Ch/DA** and **A/B**), where the common backbone is colored in cyan.

Considering only the HQSAR contribution maps of **24** (most active, Figure 3), both models are able to identify fragments which increase the biological activity, since in both models there are fragments colored in yellow and green. However, in the case of **6** (least active, Figure 3), only the **A/B/C/Ch/DA** model is able to identify fragments that decrease the activity, since only in this model is there at least one fragment colored in red. On the other hand, the **A/B** model of **6** (Figure 3) shows only fragments colored in white (neutral contribution) and cyan (common backbone), featuring fragments without correlation with the biological activity variation. Consequently, the **A/B/C/Ch/DA** model seems to be the most able to distinguish among the most and least active compounds, and thus, it is the most useful in the medicinal chemistry context.

An additional feature, observed only in the **A/B/C/Ch/DA** model of **24** (Figure 3), is the presence of a green colored fragment that corresponds to the nitrogen atom of the thiazolyl group (R_3_ substituent, Table 7). Since only this model has the H-bond donor/acceptor (DA) fragment distinction parameter, this feature highlights the importance of this atom as an H-bond acceptor in a potential H-bonding interaction in the ligand-enzyme complex. Moreover, it also reinforces the **A/B/C/Ch/DA** model as the best model. Therefore, only this model will be discussed from this point forward.

The contribution map of **24** (Figure 3), according to the best HQSAR model, shows three substituents, namely R_1_, R_2_, and R_3_ (Table 7), which significantly influence the biological activity of this series. The benzodioxol (R_1_), methyl (R_2_), and thiazolyl (R_3_) groups are present in the most active compounds, such as 24, 26, and 27. In fact, all these groups have fragments (at least one atom) colored in green or yellow, highlighting their positive contributions to biological activity.

**Table 7:** Biological activities (IC_50_, nM) and its negative logarithmic values (pIC_50_, M) for a series of 6-arylquinazolin-4-amine derivatives. 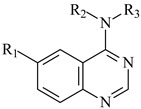

# ^a,b,c^	R_1_	R_2_	R_3_	IC_50_	pIC_50_
**1**	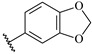	H	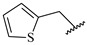	62	7.21
**2**	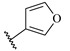	H	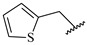	1262	5.90
**3** *	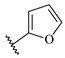	H	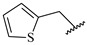	3480	5.46
**4**	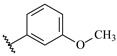	H	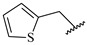	5697	5.24
**5**	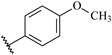	H	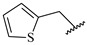	3152	5.50
**6**	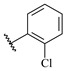	H	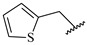	9012	5.05
**7**	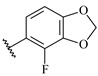	H	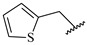	164	6.79
**8**	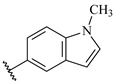	H	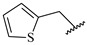	4517	5.35
**9**	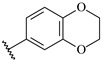	H	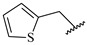	180	6.74
**10**	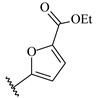	H	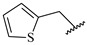	1437	5.84
**11** *	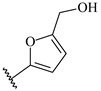	H	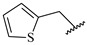	4657	5.33
**12**	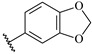	CH_3_	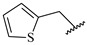	31	7.51
**13**	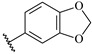	CH_2_CH_3_	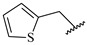	38	7.42
**14**	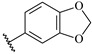	H	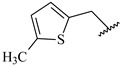	1158	5.94
**15**	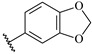	CH_3_	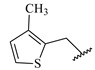	260	6.59
**16**	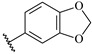	H	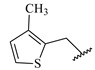	35	7.46
**17**	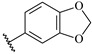	H	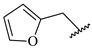	84	7.08
**18**	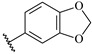	CH_3_	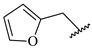	98	7.01
**19**	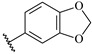	H	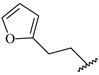	74	7.13
**20** *	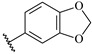	H	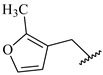	57	7.24
**21**	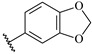	H	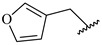	126	6.90
**22**	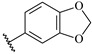	H	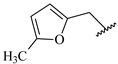	93	7.03
**23** *	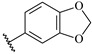	H	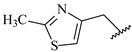	206	6.69
**24**	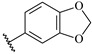	CH_3_	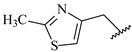	14	7.85
**25**	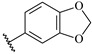	H	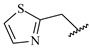	70	7.15
**26**	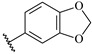	CH_3_	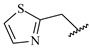	51	7.29
**27**	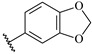	CH_3_	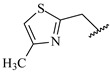	26	7.59
**28**	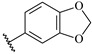	H	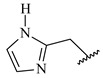	155	6.81
**29** *	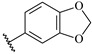	CH_3_	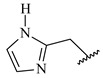	922	6.04
**30**	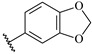	H	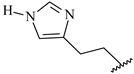	541	6.27
**31**	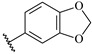	CH_3_	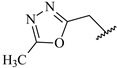	120	6.92
**32** *	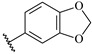	H	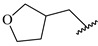	93	7.03
**33** *	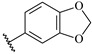	H	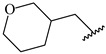	135	6.87
**34** *	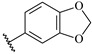	H	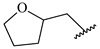	30	7.52
**35**	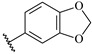	H	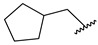	76	7.12
**36** *	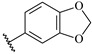	H	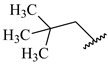	17	7.77
**37**	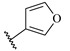	CH_3_	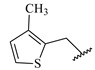	1136	5.94
**38**	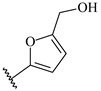	CH_3_	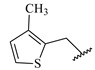	557	6.25
**39**	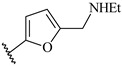	H	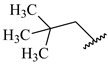	594	6.23
**40**	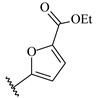	H	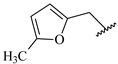	3629	5.44
**41**	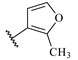	H	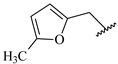	3388	5.47
**42**	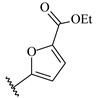	CH_3_	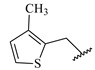	1501	5.82
**43** *	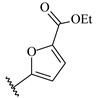	H	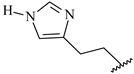	1406	5.85
**44**	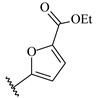	H	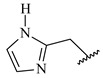	2706	5.57
**45**	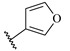	H	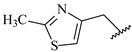	4820	5.31
**46**	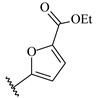	H	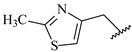	8307	5.08

^a^ Training set (36 compounds). The 10 test set compounds are marked with an asterisk (*); ^b^ Compounds **42** to **46** are from [33], and **1** to **41** are from [20]; ^c^ Compounds **32**, **33**, and **34** (all from the test set) have one chiral center and their biological activities are from their respective racemic mixture.

The contribution map of **6** (Figure 3), according to the best HQSAR model, shows one atom colored in red located on the *ortho*-chloro-phenyl group (R_1_), which is detrimental to the biological activity, probably because the chlorine atom at the *ortho* position would prevent higher co-planarity between the two aromatic groups, a feature which may be important in the ligand-protein interaction. Besides, the presence of a fragment colored in red, the lack of green or yellow colored fragments also contributes to the low activity of **6**, such as the replacement of methyl (R_2_) by hydrogen and thiazolyl (R_3_) by thiophenyl.

Some of these results are in agreement with those presented by Pan *et al.* [34] in an atom-based 3D-QSAR modeling study, using this same series of 6-arylquinazolin-4-amines. They observed that the inhibitory activity increases when R_1_ is a phenyl ring substituted with a hydrophilic and electron-withdrawing group, R_3_ is a heterocyclic ring substituted with a hydrophobic group, and the nitrogen atom of the amine group is substituted with a bulky hydrophobic group. On the other hand, the inhibitory activity decreases when R_2_ is a hydrogen atom and R_1_/R_3_ are hydrophobic groups [34].

## 3. Experimental Section

### 3.1. Chemical and Biological Data Series

The data set comprises 46 compounds from a series 6-arylquinazolin-4-amines and their biological activities, *i.e.*, the half-maximal inhibitory concentrations (IC_50_, nM), which were collected from the literature [20,33]. The IC_50_ values were expressed in negative logarithmic scale, *i.e.*, pIC_50_ (−LogIC_50_, M). Table 7 shows the chemical structures and pIC_50_ values of this series.

For the HQSAR analysis, the data set were divided in training (36 compounds, including the most and the least active compounds) and test (10 compounds) sets. The training set is used for model development and internal validation (cross-validation), while the test set is used only in the external validation of the best models. The division was not entirely random because it was necessary to ensure chemical and biological diversity for both sets. Compounds **32**, **33**, and **34**, containing one chiral center, were included in the test set because their biological activity values were from the racemic mixture. Therefore, they were modeled separately as each of the two enantiomeric forms (*R* and *S*).

### 3.2. Molecular Modeling

The chemical structures of these 46 derivatives were built up using the commercial Spartan software (version 10, Wavefunction, Inc., Irvine, CA, USA) [35]. All structures were submitted to the default systematic conformational analysis, using the AM1 semi-empirical method, available in Spartan.

### 3.3. HQSAR Model Development

HQSAR modeling was performed using the commercial SYBYL software (version 8.0, Tripos International, St. Louis, MO, USA) [36]. During the HQSAR models development, the default hologram lengths were used (53, 59, 61, 71, 83, 97, 151, 199, 257, 307, 353, and 401 bins), keeping the default fragment size (4–7 atoms). After that, the fragment size was evaluated from 2 to 12 atoms per fragment. Finally, six types of fragment distinction were combined using atoms (A), bonds (B), connectivity (C), hydrogen (H) atoms, chirality (Ch), and donor/acceptor (DA) atoms.

The HQSAR models were generated using the partial least squares (PLS) analysis, while the internal validation procedure was performed by the leave-one-out (LOO) cross-validation approach. Subsequently, the best HQSAR models were selected based on various statistical parameters, including the squared correlation coefficient (*R*^2^) and the LOO cross-validated *R*^2^ (*q*^2^) values.

In order to evaluate the risk of fortuitous correlation, the Y-randomization (also called Y-scrambling or response randomization) test, an additional validation procedure, in which the biological activity values are randomized and the HQSAR analysis is carried out again for the same training set [37] was performed.

An external validation was carried out, using the test set compounds, which were not considered for the HQSAR model development. The predictive capacity of the models was investigated by calculating the predictive *R*^2^ values (*R*^2^_pred_) values, defined according to Equation (1).

In Equation (1), SD is the sum of squared deviations between the biological activity of the test set and the mean activity of the training set molecules, and PRESS is the sum of squared deviations between the observed and the predicted activity values for every molecule in the test set [38].

Importantly, those models are based on a receptor independent QSAR method, *i.e.*, the enzyme structure was not considered, but information about the binding site of the target enzyme is available online in the Protein Data Bank (http://www.rcsb.org/), since there are crystal structures of some inhibitors bound to the same binding site of human DYRK1A [39,40,41,42]. In addition, it is also important to emphasize that user-friendly and publicly accessible web-servers pointed out in [43] are useful tools to share information with the scientific community. However, all softwares used in the current work are commercial and have patent protection, thus they could not be provided in a web-server.

## 4. Conclusions

HQSAR (2D fragment-based) models were developed for 46 6-arylquinazolin-4-amines (*N* training = 36; *N* test = 10), a series of inhibitors for DYRK1A, an enzyme associated with Alzheimer’s disease. The best model, namely **A/B/C/Ch/DA** (*q*^2^ = 0.757; SE_cv_ = 0.493; *R*^2^ = 0.937; SE = 0.251; *R*^2^pred = 0.659), contains 3–6 atoms per fragment and encodes atoms, bonds, connectivity, chirality, and donor/acceptor atoms as fragment distinctions. It presents high goodness-of-fit (*R*^2^ > 0.9), as well as high internal (*q*^2^ > 0.7) and external (*R*^2^pred > 0.5) predictive power, which indicate the reliability of the constructed model. According to the Y-randomization test (*q*^2^ ≤ 0.211), the observed correlation is not due to chance. The HQSAR colored diagrams display the contributions of the fragments in the increase or decrease of the biological activity of the compounds. The positive and negative contributions of the fragments addressed by those diagrams are in accordance with a previously performed 3D-QSAR characterization and thus may be helpful to design new 6-arylquinazolin-4-amine derivatives with enhanced DYRK1A inhibitory activity.

## References

[1] Barker W.W., Luis C.A., Kashuba A., Luis M., Harwood D.G., Loewenstein D., Waters C., Jimison P., Shepherd E., Sevush S. (2002). Relative frequencies of Alzheimer disease, Lewy body, vascular and frontotemporal dementia, and hippocampal sclerosis in the state of Florida brain bank. Alzheimer Dis. Assoc. Disord..

[2] Holtzman D.M., Morris J.C., Goate A.M. (2011). Alzheimer’s disease: The challenge of the second century. Sci. Transl. Med..

[3] Golde T.E., Eckman C.B., Younkin S.G. (2000). Biochemical detection of Aβ isoforms: Implications for pathogenesis, diagnosis and treatment of Alzheimer’s disease. Biochim. Biophys. Acta.

[4] Kayed R., Head E., Thompson J.L., McIntire T.M., Milton S.C., Cotman C.W., Glabe C.G. (2003). Common structure of soluble amyloid oligomers implies common mechanism of pathogenesis. Science.

[5] Lucin K.M., Wyss-Coray T. (2009). Immune activation in brain aging and neurodegeneration: Too much or too little?. Neuron.

[6] Forlenza O.V. (2005). Pharmacological treatment of Alzheimer’s disease (*Tratamento farmacológico da doença de Alzheimer*). Rev. Psiquiatr. Clin..

[7] Becker W., Joost H.G. (1998). Structural and functional characteristics of DYRK, a novel subfamily of protein kinases with dual specificity. Prog. Nucleic Acid Res. Mol. Biol..

[8] Park J., Song W.J., Chung K.C. (2009). Function and regulation of DYRK1A: Towards understanding Down syndrome. Cell. Mol. Life Sci..

[9] Weigel J., Gong C.X., Hwang Y.W. (2011). The role of DYRK1A in neurodegenerative diseases. Fed. Eur. Biochem. Soc..

[10] Smith B., Medda F., Gokhale V., Dunckley T., Hulme C. (2012). Recent advances in the design, synthesis and biological evaluation of selective DYRK1A inhibitors: A new avenue for a disease modifying treatment of Alzheimer’s?. ACS Chem. Neurosci..

[11] Bain J., McLauchlan H., Elliot M., Cohen P. (2003). The specificities of protein kinase inhibitors: An updtate. Biochem. J..

[12] Cozza G., Mazzorana M., Papinutto E., Bain J., Elliott M., di Maira G., Gianoncelli A., Pagano M.A., Sarno S., Ruzzene M. (2009). Quinalizarin as a potent, selective, and cell permeable inhibitor of protein kinase CK2. Biochem. J..

[13] Ahmadu A., Abdulkarim A., Grougnet R., Myrianthopoulos V., Tillequin F., Magiatis P., Skaltsounis A.-L. (2010). Two new peltogynoids from *Acacia nilotica* Delile with kinase inhibitory activity. Planta Med..

[14] Sarno S., Mazzorana M., Traynor R., Ruzzene M., Cozza G., Pagano M.A., Meggio F., Zagotto G., Battistutta R., Pinna L.A. (2012). Structural features underlying the selectivity of the kinase inhibitors NBC and dNBC: Role of a nitro group that discriminates between CK2 and DYRK1A. Cell. Mol. Life Sci..

[15] Sánchez C., Salas A.P., Braña A.F., Palomino M., Pineda-Lucena A., Carbajo R.J., Méndez C., Moris F., Sala J.A. (2009). Generation of potent and selective kinase inhibitors by combinatorial biosynthesis of glycosylated indolocarbazoles. Chem. Commun..

[16] Kim N.D., Yoon J., Kim J.H., Lee J.T., Chon Y.S., Hwang M-K., Ha I., Song W-J. (2006). Putative therapeutic agents for the learning and memory deficits of people with Down syndrome. Bioorg. Med. Chem. Lett..

[17] Echalier A., Bettayeb K., Ferandin Y., Lozach O., Clément M., Valette A., Liger F., Marquet B., Morris J.C., Endicott J.A. (2008). Meriolins (3-(pyrimidin-4-yl)-7-azaindoles): Synthesis, kinase inhibitory activity, cellular effects, and structure of CDK2/cyclin A/meriolin complex. J. Med. Chem..

[18] Giraud F., Alves G., Debiton E., Nauton L., Théry V., Durieu E., Ferandin Y., Lozach O., Meijer L., Anizon F., Pereira E. (2011). Synthesis, protein kinase inhibitory potencies, and *in vitro* antiproliferative activities of meridianin derivatives. J. Med. Chem..

[19] Neagoie C., Vedrenne E., Buron F., Mérour J.-Y., Rosca S., Bourg S., Lozach O., Meijer L., Baldeyrou B., Lansiaux A. (2012). Synthesis of chromeno[3,4-*b*]indoles as lamellarin D analogues: A novel DYRK1A inhibitor class. Eur. J. Med. Chem..

[20] Rosenthal A.S., Tanega C., Shen M., Mott B.T., Bougie J.M., Nguyen D.-T., Misteli T., Auld D.S., Maloney D.J., Thomas C.J. (2011). Potent and selective small molecule inhibitors of specific isoforms of CDC2-like kinases (CLK) and dual specificity tyrosine-phosphorylation-regulated kinases (DYRK). Bioorg. Med. Chem. Lett..

[21] Free S.M., Wilson J.W. (1964). A mathematical contribution to structure-activity studies. J. Med. Chem..

[22] Hansch C., Fujita T. (1964). ρ-σ-π Analysis. A method for the correlation of biological activity and chemical structure. J. Am. Chem. Soc..

[23] Du Q.-S., Huang R.-B., Chou K.-C. (2008). Recent advances in QSAR and their applications in predicting the activities of chemical molecules, peptides and proteins for drug design. Curr. Protein Pept. Sci..

[24] Du Q.-S., Mezey P.G., Chou K.-C. (2005). Heuristic molecular lipophilicity potential (HMLP): A 2D-QSAR study to LADH of molecular family pyrazole and derivatives. J. Comput. Chem..

[25] Du Q.-S., Huang R.-B., Wei Y.-T., Du L.-Q., Chou K.-C. (2008). Multiple field three dimensional quantitative structure-activity relationship (MF-3D-QSAR). J. Comput. Chem..

[26] Prado-Prado F.J., González-Díaz H., de la Vega O.M., Ubeira F.M., Chou K.-C. (2008). Unified QSAR approach to antimicrobials. Part 3: First multi-tasking QSAR model for input-coded prediction, structural back-projection, and complex networks clustering of antiprotozoal compounds. Bioorg. Med. Chem..

[27] Du Q.-S., Huang R.-B., Wei Y.-T., Pang Z.-W., Du L.-Q., Chou K.-C. (2009). Fragment-based quantitative structure-activity relationship (FB-QSAR) for fragment-based drug design. J. Comput. Chem..

[28] Wei H., Wang C.-H., Du Q.-S., Meng J., Chou K.-C. (2009). Investigation into adamantane-based M2 inhibitors with FB-QSAR. Med. Chem..

[29] Tong W., Lowis D.R., Perkins R., Chen Y., Welsh W.J., Godette D.W., Heritage T.W., Sheehan D.M. (1998). Evaluation of quantitative structure-activity relationship methods for large-scale prediction of chemicals binding to the estrogen receptor. J. Chem. Inf. Comput. Sci..

[30] Feher M., Ewing T. (2009). Global or local QSAR: Is there a way out?. QSAR Comb. Sci..

[31] Buchwald F., Girschick T., Seeland M., Kramer S. (2011). Using local models to improve (Q)SAR predictivity. Mol. Inform..

[32] Kruhlak N.L., Benz R.D., Zhou H., Colatsky T.J. (2012). (Q)SAR modeling and safety assessment in regulatory review. Clin. Pharm. Ther..

[33] Rosenthal A.S., Tanega C., Shen M., Mott B.T., Bougie J.M., Nguyen D.-T., Misteli T., Auld D.S., Maloney D.J., Thomas C.J. (2010). An inhibitor of the CDC2-like kinase 4 (CLK 4). Probe Reports from the NIH Molecular Libraries Program.

[34] Pan Y., Wang Y., Bryant S.H. (2013). Pharmacophore and 3D-QSAR characterization of 6-arylquinazolin-4-amines as CDC2-like kinase 4 (CLK4) and dual specificity tyrosine-phosphorylation-regulated kinase-1A (DYRK1A) inhibitors. J. Chem. Inf. Model..

[35] Dewar M.J.S., Zoebisch E.G., Healy E.F., Stewart J.J.P. (1985). Development and use of quantum mechanical molecular models. 76. AM1: A new general purpose quantum mechanical molecular model. J. Am. Chem. Soc..

[36] Tripos (2010). Sybyl 8.0.

[37] Rücker C., Rücker G., Meringer M. (2007). y-Randomization and its variants in QSPR/QSAR. J. Chem. Inf. Model..

[38] Cramer R.D., Patterson D.E., Bunce J.D. (1998). Comparative molecular field analysis (CoMFA). 1. Effect of shape on binding of steroids to carrier proteins. J. Am. Chem. Soc..

[39] Ogawa Y., Nonaka Y., Goto T., Ohnishi E., Hiramatsu T., Kii I., Yoshida M., Ikura T., Onogi H., Shibuya H. (2010). Development of a novel selective inhibitor of the Down syndrome-related kinase DYRK1A. Nat. Commun..

[40] Anderson K., Chen Y., Chen Z., Dominique R., Glenn K., He Y., Janson C., Luk K.C., Lukacs C., Polonskaia A. (2013). Pyrido(2,3-*d*)pyrimidines: Discovery and preliminary SAR of a novel series of DYRK1B and DYRK1A inhibitors. Bioorg. Med. Chem. Lett..

[41] Tahtouh T., Elkins J.M., Filippakopoulos P., Soundararajan M., Burgy G., Durieu E., Cochet C., Schmid R.S., Lo D.C., Delhommel F. (2012). Selectivity, co-crystal structures, and neuroprotective properties of leucettines, a family of protein kinase inhibitors derived from the marine sponge alkaloid leucettamine B. J. Med. Chem..

[42] Soundararajan M., Roos A.K., Savitsky P., Filippakopoulos P., Kettenbach A.N., Olsen J.V., Gerber S.A., Eswaran J., Knapp S., Elkins J.M. (2013). Structures of Down syndrome kinases, DYRKs, reveal mechanisms of kinase activation and substrate recognition. Structure.

[43] Chou K.-C., Shen H.-B. (2009). Review: Recent advances in developing web-servers for predicting protein attributes. Nat. Sci..

